# Curious Practice in a Case of Strangulated Hernia

**Published:** 1829-01-01

**Authors:** 


### XLVII.

### MIDDLESEX HOSPITAL.

STRANGULATED INGUINAL HERNIA — INTESTINE OPENED.

The following is a curious case, or rather, the surgical practice is curious.

A man, aged 42, was admitted, Oct. 2, under the care of Mr. Mayo, with strangulated inguinal, congenital hernia on the right side. He had always worn a truss, and whenever the hernia descended, found little or no difficulty in replacing it within the abdomen. His bowels had been confined for several days, and he was making a violent bodily exertion on the evening of the 1st, when the rupture came down in more than its ordinary volume. He was bled that night, and attempts at reduction made without success.

When seen by Mr. Mayo, shortly after his admission, the tumour was painful and tense, the abdomen was tender upon pressure, and a sensation of dragging was felt at the epigastrium. He had vomited once or twice, felt nausea and hiccup, had an anxious countenance and irregular pulse. An enema, the warm-bath, and the taxis, were employed, but the latter gave such pain, that the knife was had recourse to at once. The sac contained a foot of small intestine, which adhered to the neck of the sac, was dark, inflamed, and "so discoloured here and there, as to shew that gangrene was commencing."

"The adhesion of the intestine to the neck of the sack *having been broken through* with the finger, (we are told before that these were pretty firm) the stricture divided *and the intestine opened*, the opening was made fast to the divided integuments by a ligature, and the rest of the bowels returned into the belly."

The patient required bleeding next day, but otherwise his progress to recovery has been constant. The tenderness of the belly has subsided, the tongue is nearly clean, and the case will most probably end in the establishment of an artificial anus at the groin.—*Gazette*.

In cases where the gut is actually mortified, surgeons are in the habit of cutting into it, for sufficient and obvious reasons. Sir Astley Cooper observes, in his Lectures on Hernia, that—

"If the intestine be in a state of gangrene, it will have a fetid smell, the peritoneal surface will have lost its brilliancy, and be of a dark port wine colour, with greenish spots on it; it will not possess any sensibility, and will easily give way under slight pressure.

"Under *these circumstances*, the stricture should be divided in the manner I have described, after which, a free incision should be made into the gangrenous intestine, to allow of the escape of its contents, and then it should be returned to the upper part of the sac, the wound should be left open, and a poultice applied; but, if the portion of intestine which has descended be not large, it should not be disturbed from its adhesions to the sac."

In the present instance, the gut had few or none of the characters of gangrene, being merely, in the words of the report, so discoloured, as to shew that this state was *commencing*. Now, the practice appears to be singular, the incisions of the gut premature, and not exactly called for by the symptoms detailed by the reporter. The account, altogether, is imperfect and confused, and we trust that Mr. Mayo will clear up the doubts its perusal unavoidably tends to produce. As it stands, the *rationale* of the practice is deficient, but Mr. Mayo, we are sure, will be able to explain in a satisfactory manner, the grounds on which he acted.

